# Association between GnRH analogue use and atopic diseases in patients with prostate cancer: A population-based retrospective cohort study

**DOI:** 10.1371/journal.pone.0266771

**Published:** 2022-04-11

**Authors:** Sheng-Feng Lin, Hsiu-Chen Lin, Mei-Yu Lee, Joseph Jordan Keller, Li-Hsuan Wang

**Affiliations:** 1 Department of Public Health, School of Medicine, College of Medicine, Taipei Medical University, Taipei, Taiwan; 2 School of Public Health, College of Public Health, Taipei Medical University, Taipei, Taiwan; 3 Department of Emergency Medicine, Taipei Medical University Hospital, Taipei, Taiwan; 4 Department of Pediatrics, School of Medicine, Taipei Medical University, Taipei, Taiwan; 5 Department of Clinical Pathology, Taipei Medical University Hospital, Taipei, Taiwan; 6 School of Pharmacy, College of Pharmacy, Taipei Medical University, Taipei, Taiwan; 7 College of Medicine, Ohio State University, Columbus, Ohio, United States of America; 8 Department of Pharmacy, Taipei Medical University Hospital, Taipei, Taiwan; Seoul National University College of Medicine, REPUBLIC OF KOREA

## Abstract

**Purpose:**

Gonadotropin-releasing hormone (GnRH) analogues reduce testosterone levels to castration levels in patients with prostate cancer. However, the role of testosterone in atopic diseases has remained undefined. We aimed to investigate this role.

**Materials and methods:**

This retrospective cohort study was conducted using the National Health Insurance Research Database (NHIRD). Patients with prostate cancer were categorized into two groups according to whether they received GnRH analogue treatment (study group I) or not (study group II), and men without prostate cancer and with no GnRH analogue use were defined to comprise the comparison group after their ages and index years were matched with group II. Cox proportional hazard models were used to assess the hazard ratio (HR) of atopic diseases.

**Results:**

Group I, group II, and the comparison group comprised 663, 2,172, and 8,688 individuals, respectively. Group I had a significantly lower risk of atopic diseases (adjusted HR: 0.66, 95% CI, 0.49–0.89, *p* < 0.01) than did group II. A reduced risk of atopic diseases was found when GnRH analogues were prescribed for 2 months (adjusted HR 0.53, 95% CI, 0.29–0.97, *p* = 0.04) and 2–14 months (adjusted HR 0.66, 95% CI, 0.49–0.89, *p* = 0.007). No significant difference in the risk of atopic diseases between group II and the comparison group was observed.

**Conclusions:**

A decreased risk of atopic diseases was observed in patients with prostate cancer treated with GnRH analogues. Further studies are warranted to verify the association between testosterone levels and atopic diseases.

## Introduction

Atopic diseases, including atopic dermatitis, allergic rhinitis, and asthma, share the common pathogenetic factors of increased T helper 2 (Th2) cytokine levels and cell responses [1–5]. The prevalence of atopic diseases, especially asthma, are consistently predominant in boys and women [6–8]. Moreover, androgens have been implicated as exerting immunomodulating effects in both animal [9, 10] and human studies [11, 12]. Increases in the Th1-biased phenotype and the number of T cells were observed in patients with prostate cancer on androgen-deprivation therapy (ADT) [13, 14]. Androgens have been known to have the protective role in inflammatory diseases and to suppress Th2 activities [15]. A large population-based cross-sectional study [16] by accessing the UK Biobank found high levels of serum testosterone was negatively associated with asthma. However, some studies [17–19] have reported androgens may negatively contribute to atopic diseases. Androgens may worsen the atopic dermatitis by impairing the skin barrier [17, 19, 20] and may aggravate asthma by modifying lung inflammation and macrophage polarization [18].

Patients with prostate cancer in an advanced stage or with recurrent prostate cancer at any stage benefit from ADT with gonadotropin-releasing hormone (GnRH) analogues (GnRH agonists or antagonists), which lower androgen levels to castration levels [21, 22]. Although GnRH agonists cause a rapid increase and then sharp decrease in testosterone levels, GnRH antagonists work by directly reducing testosterone levels [23]. However, evidence on the immunomodulating effect of androgens has mainly been derived from animal studies [9–11]. Whether the reduction in testosterone levels has beneficial effects in patients with atopic diseases has yet to be determined. We aimed to use nationwide cohort data to investigate the risk that newly diagnosed patients with prostate cancer have for developing atopic diseases after they receive ADT with GnRH analogue treatment.

## Materials and methods

### Study population

This was a retrospective cohort study. Patients with a new diagnosis of prostate cancer from January 1, 2001, to December 31, 2012, were enrolled. The data used in this study were obtained from the National Health Insurance Research Database (NHIRD), which registers the health information for >99.9% of residents in Taiwan, and can be obtained through formal requests submitted to the Taiwan Health and Welfare Data Science Center (HWDC). NHIRD data on a subpopulation, comprising two million people who were randomly sampled from the 20 million people living in Taiwan, were used in this study. Patients with prostate cancer who were aged <20 years or who received GnRH analogues before being diagnosed with prostate cancer were excluded. Of these patients, those with prostate cancer were categorized into the following two groups: (1) treatment with GnRH analogues (group I), and (2) treatment without GnRH analogues (group II). The comparison group was defined to comprise men without prostate cancer or GnRH analogue use after matching for age and index years at a 1:4 ratio. All groups (groups I and II and the comparison group) were followed up for 3 years. This study was approved by the Joint Institutional Review Board of Taipei Medical University (reference number: N20176020). Because all NHIRD data are delinked and deidentified, the requirement for informed consent was waived.

### Definition

Patients with prostate cancer were defined as those having two or more diagnosis codes (under Code 185) in the International Classification of Diseases, Ninth Revision, Clinical Modification (ICD-9-CM). The baseline characteristics of the two study groups and the comparison group, such as hypertension, diabetes mellitus, hyperlipidemia, and gastroesophageal reflux disease (GERD), were also defined using the ICD-9-CM. Personal income was classified into five levels according to the individual’s monthly insurable wage. The air pollution levels of particle matter (PM) 2.5 were classified into three levels: safe (<15 μg/m^3^), above the threshold (15–25 μg/m^3^), and hazardous (>25 μg/m^3^), according to data from the public database of the Taiwan Environmental Protection Administration.

Agents of the following GnRH analogues were used for analysis: buserelin, leuprorelin, goserelin, triptorelin, and degarelix. Patients were classified by duration of treatment using a one-month equivalent dose.

### Confounding factors

The following comorbidities were considered to be confounders of the association between GnRH analogues and atopic diseases: hypertension, diabetes mellitus, hyperlipidemia, GERD, and a habit of tobacco use. Patients’ age and medication history of statin, acetaminophen, and oral corticosteroids were assessed for the study’s three groups.

### Outcome measure

The primary outcome measure of this study was the development of atopic diseases, including allergic dermatitis, allergic rhinitis, and asthma. The corresponding ICD-9-CM codes used to assess the development of these diseases were 691.8, 477, and 493, respectively. To further investigate whether GnRH treatment duration affected the risk for atopic diseases in patients with prostate cancer, we measured the long-term effects of GnRH analogues on atopic disease occurrence as the secondary outcome.

### Statistical analysis

Between the two groups, continuous variables were compared using Student’s *t* test and categorical variables were compared using Pearson’s chi-square test. Each group was followed up for 3 years. Cox proportional hazard models were employed, in which the occurrence of atopic disease was the dependent variable and the following were independent variables: prescription of GnRH analogues and the covariates of age; hypertension; diabetes mellitus; hyperlipidemia; GERD; tobacco use; medications of stain, acetaminophen, and oral corticosteroids; air pollution levels; and income. Statistical significance was indicated if *p* < 0.05 (two-tailed). SAS 9.4 software (SAS Institute Inc., Cary, NC, USA) was used for analysis.

## Results

### Study population

**Fig 1:**
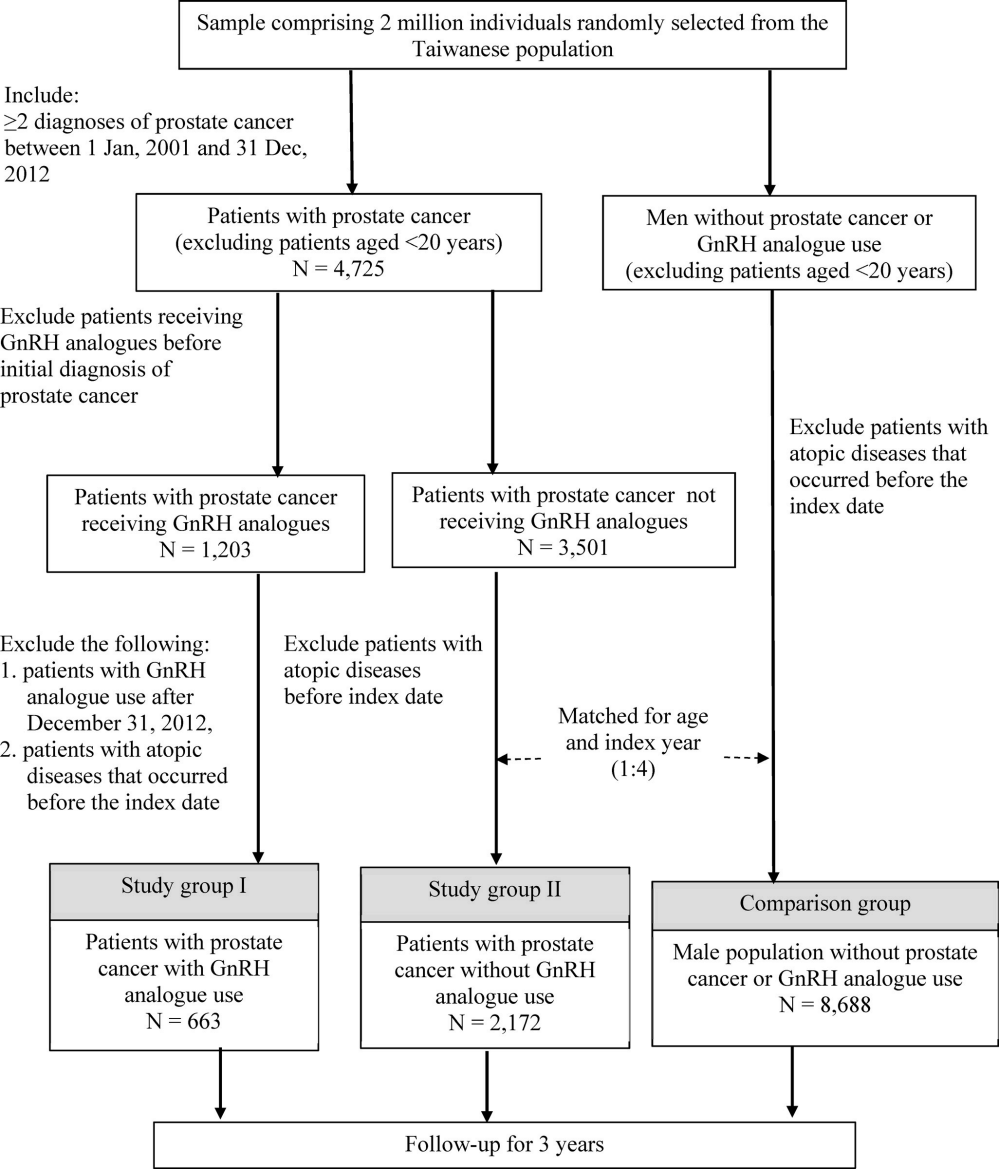
Flow diagram of participant recruitment.

A total of 663 and 2,172 patients with prostate cancer were identified and assigned to groups I and II, respectively. They were classified according to whether they were treated with or without GnRH analogues into groups I and II, respectively. An additional 8,688 patients were placed in the comparison group. Fig 1 presents a flowchart of this process. Table 1 presents the differences in the baseline characteristics between group I and group II and between group II and the comparison group. Compared with the comparison group, both groups I and II had higher proportions of medical comorbidities of hypertension, diabetes mellitus, hyperlipidemia, and GERD and statin and non-steroidal anti-inflammatory drug (NSAID) use. The air pollution levels and incomes of all three groups were similar.

**Table 1:** Baseline characteristics of patients with prostate cancer and matched cohort.

Variables	Study group		Comparison group	*P-value* ^*a*^	*P-value* ^*b*^
	Patients with prostate cancer (2001–2012)		Without prostate cancer or GnRH analogues use male population		
	With GnRH analogues (I)	Without GnRH analogues (II)			
	N = 663	N = 2,172	N = 8,688		
**Age, years (mean ± SD)**	71.91 ± 10.14	68.40 ± 12.47	68.42 ± 12.50	<0.0001	0.954
**Hypertension (n, %)**	383 (57.77)	1,110 (51.10)	3,845 (44.26)	0.003	<0.0001
**Diabetes mellitus (n, %)**	201 (30.32)	570 (26.24)	1,988 (22.88)	0.039	< 0.001
**Hyperlipidemia (n, %)**	252 (38.01)	728 (33.52)	2,216 (25.51)	0.033	<0.0001
**GERD (n, %)**	68 (10.26)	118 (5.43)	288 (3.31)	<0.0001	<0.0001
**Tobacco use (n, %)**	12 (1.81)	23 (1.06)	133 (1.53)	0.125	0.098
**Statin (n, %)**	136 (20.51)	344 (15.84)	1,091 (12.56)	0.005	<0.0001
**Acetaminophen (n, %)**	253 (38.16)	512 (23.57)	2,091 (24.07)	<0.0001	0.629
**NSAIDs (n, %)**	398 (60.03)	975 (44.89)	3,535 (40.69)	<0.0001	< 0.001
**Oral corticosteroids (n, %)**	44 (6.64)	69 (3.18)	349 (4.02)	<0.0001	0.069
**Air pollution levels of PM 2.5** ^**c**^					
**Safe**	6 (0.90)	17 (0.78)	127 (1.46)	0.759	0.013
**Above target**	44 (6.64)	100 (4.60)	576 (6.63)	0.037	<0.001
**Hazardous**	613 (92.46)	2,055 (94.61)	7,985 (91.91)	0.039	<0.0001
**Incomes (monthly insurable wages in NT$)** ^**d**^					
**Dependent**	7 (1.06)	49 (2.26)	219 (2.52)	0.052	0.477
**≦ 20,100**	401 (60.48)	1,198 (55.16)	5,256 (60.50)	0.016	<0.0001
**20,101–40,100**	201 (30.32)	541 (24.91)	2,423 (27.89)	0.006	0.005
**40,101–60,800**	39 (5.88)	228 (10.50)	561 (6.46)	<0.0001	<0.0001
**> 60,800**	15 (2.26)	156 (7.18)	229 (2.64)	< 0.001	<0.0001

GnRH, gonadotropin-releasing hormone; SD, standard deviation; GERD, gastroesophageal reflux disease; NSAIDs, non-steroidal anti-inflammatory drugs; NT$, New Taiwan dollar.

^a^ *P* value between study group I and study group II.

^b^ *P* value between study group II and comparison group.

^c^ Air pollution level determined by the 7-year average (2006–2012) of particulate matter < 2.5μm (PM 2.5); safe: ≤ 15 μg/m^3^, above target: >15 μg/m^3^ and ≤25 μg/m^3^, hazardous: >25 μg/m^3^.

^d^ US$1 = NT$28.8 (March 2018).

### Risk of atopic diseases

**Fig 2:**
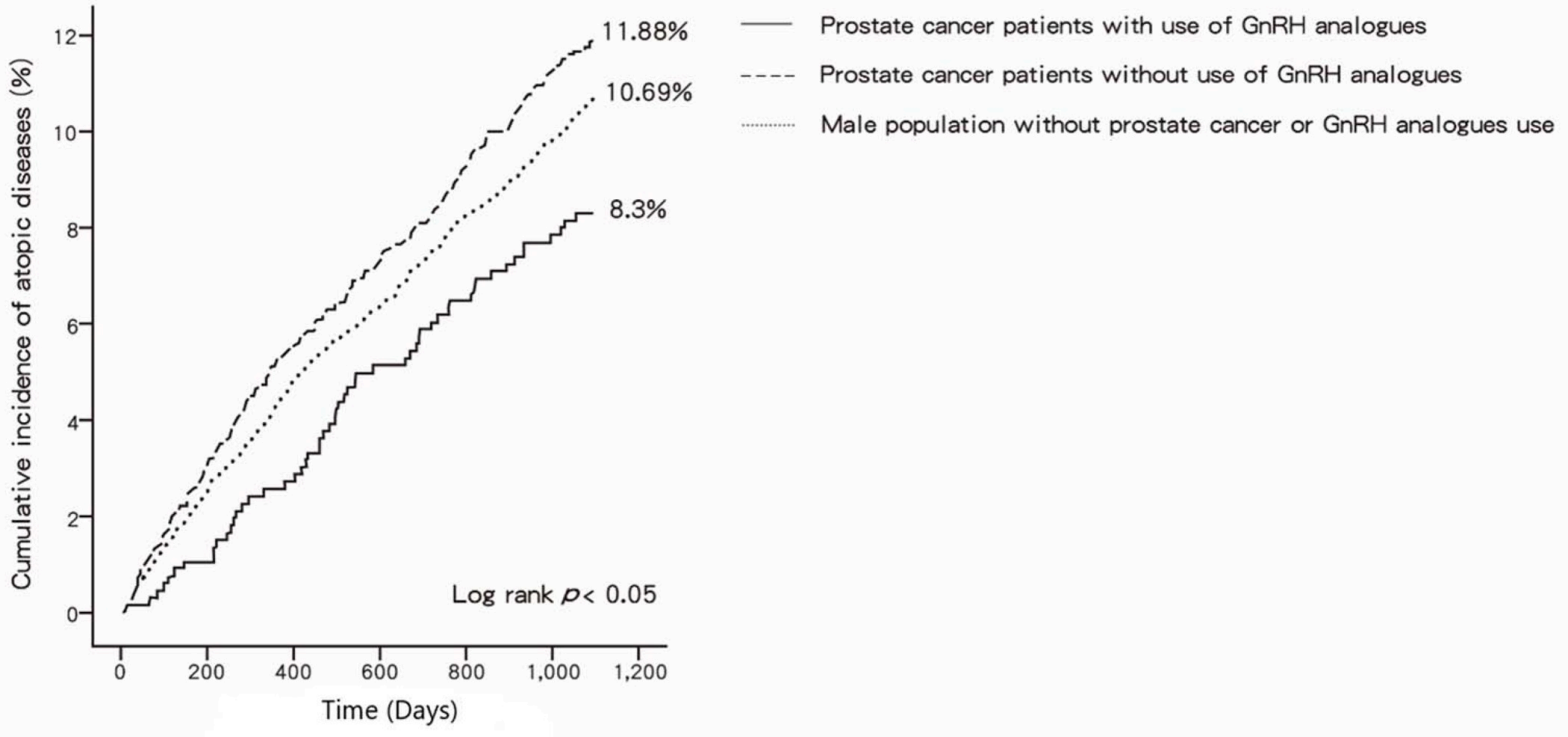
Cumulative incidence of atopic diseases.

The risk for atopic diseases during the 3-year follow-up period was assessed (Table 2). Group I had a lower risk of atopic diseases than group II did (adjusted HR: 0.66, 95% CI: 0.49–0.89, *p* < 0.01). Group I also had a lower risk for atopic diseases than did the comparison group (adjusted HR: 0.72, 95% CI: 0.54–0.94, *p* < 0.05). However, no significant difference in the risk of atopic diseases was found between group II and the comparison groups. Additionally, the following characteristics and behaviors had no association with the risk of developing atopic diseases: age, hypertension, diabetes mellitus, hyperlipidemia, GERD, tobacco use, stain, acetaminophen, oral corticosteroids, air pollution level, and income. Only NSAID use was associated with the risk of developing atopic diseases (Table 3). The Kaplan–Meier curves for patients with prostate cancer treated with or without GnRH analogues (groups I and II) and the comparison group are presented in Fig 2. A significant reduction in the occurrence of atopic diseases was found in group I patients with prostate cancer on GnRH analogues; the result of the log rank test was significant.

**Table 2:** Hazard ratios for atopic diseases in patients with prostate cancer during 3-year follow-up period.

Outcome	Patients with prostate cancer		Without prostate cancer or GnRH analogue use in male population
	With GnRH analogues (I)	Without GnRH analogues (II)	
	N = 663	N = 2,172	N = 8,688
Atopic diseases (n, %)	55 (8.30%)	258 (11.88%)	929 (10.69%)
Crude HR (95% CI)	0.68 (0.51–0.91)*	1	─
Adjusted HR (95% CI)	0.66 (0.49–0.89)**	1	─
Crude HR (95% CI)	0.77 (0.58–1.00)	1.12 (0.98–1.29)	1
Adjusted HR (95% CI)	0.72 (0.54–0.94)*	1.12 (0.98–1.29)	1
Onset time (days, mean ± SD)	522.55 ± 286.27	482.08 ± 321.33	501.49 ± 325.42

SD, standard deviation; HR, hazard ratio; CI, confidence interval.

**p < 0.01

*p < 0.05.

Hazard ratios were adjusted for age, hypertension, diabetes mellitus, hyperlipidemia, gastroesophageal reflux disease, mycoplasma pneumoniae infection, alcohol dependence syndrome, tobacco use disorder, obesity, statin, acetaminophen, non-steroidal anti-inflammatory drugs, oral corticosteroids, air pollution level, and income.

**Table 3:** Multivariate analysis of the potential risk factors of atopic diseases.

	Crude HR	95% CI	*p*	Adjusted HR	95% CI	*p*
Age	1.01	0.99–1.02	0.291	1.00	0.99–1.02	0.428
Hypertension	1.08	0.87–1.35	0.495	1.05	0.83–1.34	0.681
Diabetes mellitus	0.97	0.76–1.25	0.840	0.98	0.75–1.28	0.872
Hyperlipidemia	0.87	0.69–1.11	0.258	0.85	0.64–1.14	0.280
GERD	0.86	0.53–1.38	0.525	0.91	0.56–1.47	0.684
Tobacco use	0.51	0.13–2.03	0.337	0.55	0.14–2.23	0.406
Statin	0.91	0.67–1.24	0.553	0.94	0.65–1.35	0.735
Acetaminophen	1.12	0.88–1.43	0.365	1.06	0.81–1.38	0.682
NSAIDs	1.29	1.03–1.61	0.027	1.37	1.06–1.76	0.017
Oral corticosteroids	0.87	0.48–1.59	0.656	0.81	0.44–1.49	0.496
Air pollution level of PM 2.5						
Safe	0.76	0.19–3.05	0.698	1.20		
Above target	0.80	0.46–1.39	0.431		0.77–1.86	0.417
Hazardous	1.26	0.75–2.12	0.378			
Income†						
Dependent	1.14	0.54–2.41	0.736			
≤20,100	1.12	0.89–1.40	0.339			
20,101–40,100	0.86	0.67–1.12	0.273	1.01	0.89–1.15	0.857
40,101–60,800	0.83	0.55–1.25	0.364			
>60,800	1.23	0.80–1.88	0.340			

HR, hazard ratio; CI, confidence interval.

Hazard ratios were adjusted for age, hypertension, diabetes mellitus, hyperlipidemia, gastroesophageal reflux disease, mycoplasma pneumoniae infection, alcohol dependence syndrome, tobacco use disorder, obesity, statin, acetaminophen, non-steroidal anti-inflammatory drugs, oral corticosteroids, air pollution level, and income.

†monthly insurable wage was expressed in NT dollars.

### Accumulative exposure time of GnRH analogue use

After 1 month of GnRH analogue use, the risk of developing atopic diseases was not significantly reduced (adjusted HR: 0.56, 95% CI: 0.25–1.26, *p* = 0.161). A reduced risk of atopic diseases was evident in men undergoing GnRH analogue therapy at a prescription duration of 2 months (adjusted HR: 0.53, 95% CI: 0.29–0.97, *p* = 0.040) and 2–14 months (adjusted HR: 0.66, 95% CI: 0.49–0.89, *p* = 0.007) (Table 4). No statistical difference between the duration of GnRH analogue use and the magnitude of risk was observed in trend analysis (*p* = 0.595).

**Table 4:** Adjusted association between accumulative exposure time and atopic diseases among men receiving GnRH analogues.

Cumulative period of exposure to GnRH analogues (months)^†^	Atopic diseases		
	Adjusted HR	95% CI	*p*
**None**	Ref	**--**	***--***
**<1**	0.56	0.25–1.26	0.161
**2**	0.53	0.29–0.97	0.040
**2–14**	0.66	0.49–0.89	0.007

HR, hazard ratio; CI, confidence interval.

Cox proportional hazards model was used to adjust for age, hypertension, diabetes mellitus, hyperlipidemia, gastroesophageal reflux disease, mycoplasma pneumoniae infection, alcohol dependence syndrome, tobacco use disorder, obesity, statin, acetaminophen, non-steroidal anti-inflammatory drugs, oral corticosteroids, air pollution level, and income.

†Duration of treatment estimated by the sum of 1-month equivalent doses.

## Discussion

Our study results indicated that prostate cancer did not contribute to the risk of developing atopic disease (study group II vs. comparison group). Patients with prostate cancer treated with GnRH analogues had a decreased risk of atopic disease (study group II vs. group I). This suggests an association between GnRH analogue use and decreased occurrence of atopic diseases. A reduced risk of atopic diseases was evident in men who were on GnRH analogue therapy for a prescribed duration of more than 2 months.

Generally, testosterone has been considered to act as immunosuppressant, and earlier studies have found low testosterone levels in atopic diseases patients, including atopic dermatitis [24], asthma alone and asthma with allergic rhinitis [25]. These studies support low levels of testosterone may be associated with the presence of atopic diseases. However, in those studies, the testosterone levels were much higher (< 50 ng/dL or < 1.7 nmol/L) than the castration level of the population in these study. Furthermore, it was found that testosterone could potentially have a negative influence on the development of atopic diseases through impairing skin integrity [17–19]. It is likely that the extremely low concentrations of testosterone may contribute to this study’s unconventional results.

The immunomodulation effect of androgen deprivation may explain the association between GnRH analogue use and the reduced occurrence of atopic diseases. First, the androgens of testosterone and dihydrotestosterone (DHT) profoundly affect the immune system [11]. The androgen receptor is expressed by the thymus [26]. Furthermore, testosterone or DHT treatment is associated with a decrease in thymus size [27, 28], whereas increased thymus size and weight and the promotion of thymopoiesis are found in castrated animals [29] and humans [30]. Second, animal studies found that the use of GnRH analogues increased the number of T cells [31, 32], increased the levels of cytokine interferon-γ for Th1 stimulation [11, 31], and decreased the levels of interleukin (IL)-4 [33], IL-10 [33], and IL-6 [34] for stimulating the Th2 immune response. The Th2-based peripheral immune reaction has been recognized as an important pathogenetic factor for atopic diseases [1–5]. Because androgen-deprivation disrupts the balance between Th1 and Th2, the Th2-biased immune reaction for atopic diseases should be reduced in men with GnRH analogues.

The strengths of this population-based, retrospective cohort study are as follows, first, its nationwide sampling acquired adequate sample size of patients with prostate cancer treated with GnRH analogues. As this study was conducted using NHIRD with a coverage rate of over 99% of Taiwan population, it demonstrated high representative of overall population. Second, careful inclusion criteria and exclusion criteria were applied to this study to avoid biased results. For instance, only naïve patients with no previous exposure of GnRH analogues prior to enrollment were included in this study to avert interference of previous treatment. Moreover, patients with history of atopic diseases were excluded in this study to ensure the association between the exposure and outcome. Third, for control of confounding variables, this study has taken not only clinical factors, but also social and environmental factors into consideration, including air pollution levels and income. This study used PM 2.5 as the indicator for air pollution levels instead of geographic regions or population to truly reflect the extent of air pollution. Lastly, the disease code for prostate cancer in the NHIRD is also validated in the Taiwan National Cancer Registry [35]. In addition, this study determined the occurrence of atopic diseases as more than twice disease codes to increase positive predictive value and decrease the possibility of false positive. The calculation of atopic diseases occurrence was originated from diagnoses record on different dates or by different physicians to ensure the accuracy of the occurrence.

This study contains several limitations as well. First, as the average age of study population in this study was approximately 70 years old, the results of this study may not be generalized to younger prostate cancer patients treated with GnRH analogues. Second, since serum testosterone and DHT levels were unavailable in the NHIRD, this study cannot determine whether linear correlation existed between testosterone and DHT levels and atopic diseases occurrence. Third, due to lack of information in NHIRD, there were some confounders that cannot be controlled in this study, including genes, environmental allergens and dietary habits. However, according to epidemiological studies, the aforementioned confounders had more impact on the occurrence of atopic diseases in children than in adults. As this study aimed to observe the occurrence of atopic diseases in adult population, the residual effects of those confounders should be small.

## Conclusion

Patients with prostate cancer on ADT with GnRH analogues exhibited a significantly reduced risk of incident atopic diseases over a 2-month treatment period. Our results suggest that androgens play a role in the development of atopic diseases. Additionally, we provided a direct safety profile of atopic diseases when treating patients with prostate cancer with GnRH analogues. Thus, further studies are warranted to better elucidate the association between testosterone levels and atopic diseases.
